# Sugar-sweetened beverage consumption and breast composition in a longitudinal study of Chilean girls

**DOI:** 10.1186/s13058-021-01495-8

**Published:** 2022-01-08

**Authors:** Lara Yoon, Camila Corvalán, Ana Pereira, John Shepherd, Karin B. Michels

**Affiliations:** 1 Department of Epidemiology, Fielding School of Public Health, University of California, Los Angeles, 650 Charles Young Drive South, Los Angeles, CA 90095 USA; 2 Institute of Nutrition and Food Technology, University of Chile, Santiago, Chile; 3 Epidemiology and Population Sciences in the Pacific Program, University of Hawaii Cancer Center, Honolulu, HI USA; 4 Institute for Prevention and Cancer Epidemiology, Faculty of Medicine and Medical Center, University of Freiburg, Freiburg, Germany

**Keywords:** Nutrition, Breast density, Breast cancer, Sugar-sweetened beverages

## Abstract

**Background:**

Frequent sugar-sweetened beverage (SSB) intake has been associated with indirect markers of breast cancer risk, such as weight gain in adolescents and early menarche. How SSB intake relates to breast composition in adolescent girls has not been explored.

**Methods:**

We evaluated the association between prospective intake of SSB and breast density in a cohort of 374 adolescent girls participating in the Growth and Obesity Cohort Study in Santiago, Chile. Multivariable linear regression models were used to analyze the association between average daily SSB intake quartiles and breast composition (absolute fibroglandular volume [aFGV], percent fibroglandular volume [%FGV], total breast volume [tBV]). Models were adjusted for potential confounding by BMI Z-score, age, daily energy intake (g/day), maternal education, hours of daily television watching after school, dairy intake (g/day), meat intake (g/day), waist circumference, and menarche. To examine the sensitivity of the association to the number of dietary recalls for each girl, analyses were further stratified by girls with one dietary recall and girls with > one dietary recall.

**Results:**

A total of 881 dietary recalls were available for 374 girls prior to the breast density assessment. More than 60% of the cohort had > one dietary recall available. In multivariable analyses, we found no association between SSB intake quartile and aFGV (Q2 vs Q1 β: − 5.4, 95% CI − 15.1, 4.4; Q3 vs Q1 β: 1.3, 95% CI − 8.6, 11.3; Q4 vs Q1 β: 3.0, 95% CI − 7.1, 13). No associations were noted for %FGV and tBV. Among girls with at least one dietary recall, we found no significant associations between SSB intake quartiles and %FGV, aFGV, or tBV.

**Conclusion:**

Overall, we observed no evidence that SSB intake was associated with breast density in adolescent Chilean girls.

**Supplementary Information:**

The online version contains supplementary material available at 10.1186/s13058-021-01495-8.

## Background

Percent breast density, assessed as the proportion of fibroglandular tissue in the breast, is a strong and consistent biomarker of breast cancer risk in adult women [1]. More than 40 studies have assessed the association between breast cancer risk and increased breast density, with most reporting a two- to five-fold increase in risk for women with the densest breasts compared to those with the least dense breasts [2]. The majority of studies evaluating breast density have been based on radiographically assessed density in adult women. Yet, breast development begins during puberty, and adolescent breast composition may influence later-life breast cancer risk [3, 4]. Understanding factors associated with adolescent breast development during windows of heightened susceptibility such as puberty may be vital to understanding adult breast cancer risk. Rapid breast cell proliferation and lobular structure formation occur during the pubertal window; environmental exposures occurring during this time may have outsized influence on dense breast development [5].

Breast density is considered potentially modifiable [6, 7], and several studies have examined the influence of diet on adult breast density [8–12]. Of particular interest is sugar consumption, which has increased dramatically around the globe and has been associated with a number of negative health outcomes, including diabetes, obesity, and cancer [13, 14]. Excessive sugar consumption induces DNA damage, increases inflammation, and increases insulin production, all of which might increase cellular proliferation in breast tissues and thus increase breast density and risk of breast carcinogenesis [15, 16]. Moreover, excessive sugar consumption in the form of sugar-sweetened beverages (SSBs) is associated with increases in total caloric intake and promotion of visceral adiposity, resulting in increased weight gain and obesity risk [17]. Insulin resistance, highly prevalent among overweight and obese adolescents and adults, is positively correlated with insulin-like growth factor-1 (IGF-1), an established breast mitogen positively associated with mammographic breast density and breast cancer risk among premenopausal women [18–22]. Recently, ultra-processed foods (UPFs)-of which SSBs are a major component—have also been related to breast cancer risk, acting through hypothesized mechanisms involving higher glycemic response or inflammation due to chemical additives [23]. Altogether, this suggests that intake of SSBs may influence breast density and breast cancer risk through several pathways. Only one study has examined the association of SSBs to adult mammographic density: in a Canadian cohort, mean absolute density was higher among women in the highest quartiles of SSB intake compared to those in the lowest [10].

No studies have examined the impact of SSB intake on breast composition during adolescence. The primary hesitation in studying breast density in younger women and girls is the reluctance to expose them to radiation and discomfort during a mammographic imaging assessment [24]. Other methods for measuring breast density, including magnetic resonance imaging (MRI), have been used but cost is prohibitive on a larger scale [25]. The dual-energy X-ray absorptiometry (DXA) method to assess breast density (via fibroglandular volume) has been developed and validated for use in young girls [26]. The DXA assessment has been successfully used in epidemiologic studies to associate breast density with exposures during the childhood window of susceptibility, including intake of specific nutrients [27, 28]. Additional research is needed to understand the effects of other childhood exposures on breast development.

We have previously found an association between more frequent intake of sweetened artificially-flavored milk-based drinks and higher breast density among Chilean girls participating in the Growth and Obesity Cohort Study (GOCS) [27]. The relation was not present for plain milk, suggesting that added sugar might be driving the association and necessitating further study of beverages of any type with added sugar. We hypothesized that higher SSB intake is associated with higher absolute breast density and percent breast density, when accounting for body composition, among adolescents. Therefore, we examined the association between SSB intake and breast composition in a large, prospective cohort of adolescent girls in Santiago, Chile.

## Methods

### Study population

The ongoing longitudinal GOCS study was initiated in 2006 and is comprised of children born in 2002–2003 from six counties of Santiago, Chile [29]. Children included in the initial cohort met the following criteria: (i) aged 2.6–4 years in 2006, (ii) attendance at a low- or middle-low-income nursery belonging to the National Association of Dare Care Centers (JUNJI) in 2006, (iii) full-term (37–42 week) singleton birth with birth weight > 2500 g, (iv) evidence of prior year enrollment in a JUNJI center, and (v) free from physical and psychological conditions affecting growth (e.g., skin burns, brain tumor, hyperthyroidism). Of the 1498 eligible participants, 80% (1190) agreed to participate and enroll in the study; 515 were girls. Children included in the cohort visited the Institute of Nutrition and Food Technology (INTA) Health Clinic roughly once per year from 2006 to 2010; in 2011, the visit frequency increased to twice per year in order to more precisely capture Tanner (pubertal) staging. At the Health Clinic, participants received a physical exam that included assessment of anthropometric measures (weight, height, body mass index, waist circumference) and pubertal development (Tanner stage, breast composition, age at menarche), in addition to collection of biological specimen and dietary nutrition intake via 24-h (24H) recall.

Of the 515 girls who enrolled in the cohort, 460 had active follow-up data through Tanner stage 4 or 5. We excluded 56 girls with missing breast composition measurements and an additional 86 girls with no prospective dietary data. The final analytic cohort included 374 girls.

### Assessment of breast tissue density

Tanner assessments were performed approximately every 6 months by two dietitians trained under the supervision of a pediatric endocrinologist. Breast composition was measured when the girls first reached Tanner stage 4 (“Tanner 4”). In the subset of cases where the girl had progressed past Tanner 4 at the clinic visit, breast density was assessed at Tanner 5 (n = 81). DXA was used to assess the volume of dense breast tissue (fibroglandular volume [FGV]) in a process developed by Shepherd et al. [30], using the software version 5 [31]. In short, the left and right breast were scanned using Prodigy DXA system software (version 13.6, series 200674; GE Healthcare). Quality control and calibration were performed on a regular basis and evaluated by Dr. Shepherd. The DXA approach has been validated and has high precision for evaluating adolescent breast density [26, 31]. Absolute FGV (aFGV; cm^3^) and total volume (tBV; cm^3^) were defined following a two-compartment model of adipose and fibroglandular tissue [30]. Percent FGV (%FGV; %) was defined as the proportion fibroglandular volume relative to total volume times 100. Measurements from the left and right breast were averaged to obtain a value for aFGV, tBV, and %FGV for all analyses. We included %FGV and aFGV as measures of breast density and tBV as a measure of breast size in the analyses. All breast composition metrics are reported as continuous values.

### Assessment of dietary intake

Biannual 24H dietary recalls were collected from April 2013 through December 2015 by two dietitians using the USDA multiple-pass method [32]. At each clinic visit, trained dietitians asked the girls to recall all the foods and beverages consumed during the previous day. Visual (National Dietary Survey food atlas, [33]) and verbal cues were provided to help the girls recall the type and portion size of foods they had consumed in the 24 h prior, as well as cooking method, mealtime, location, and brand. Nutrient information was obtained by mapping Chilean foods to the USDA Food and Nutrient Database for Dietary following a harmonization process [34]. A total of 1457 dietary recalls were available for the 460 girls in the cohort with a breast composition assessment. To ensure that we appropriately characterized dietary exposure as occurring prior to the breast outcomes, we dropped 576 recalls that were collected after the breast composition assessment at the individual level. A total of 881 prospective dietary recalls were available for the study sample; 79% were collected on a weekday, 17%, on a weekend, and 4%, on a holiday. The majority of recalls were completed in the months of May, June, and July; the fewest number of recalls were completed in March, September, and November. The maximum follow-up period between 24H dietary recall and breast outcome assessment was 35 months; the minimum, < one month. The average follow-up time for the 24H recalls was 7.5 months; among girls with only one recall, follow-up time did not exceed 7 months. Girls with only one recall were more likely to be younger (mean age at recall: 10.8 years) than girls with more than one recall (mean age at recall: 11.3 years).

Our primary exposure was average daily intake of sugar-sweetened beverages (mL/day) from 2013 (mean age = 10.8) through 2015 (mean age = 12.4). Sugar-sweetened beverages were defined as any beverage with added sugar including flavored water, energy and sport drinks, full-sugar (i.e., non-diet) sodas, fruit and vegetable juice drinks with added sugar, coffee and tea with added sugar, dairy-based beverages and dairy substitutes with added sugar, and powdered drinks with added sugar (e.g., Nesquik chocolate). We have described the contribution of added sugar to these products in previous studies [35, 36]. For each girl, sugar-sweetened beverage intake was averaged over her follow-up period to reduce random-measurement error (mean = 2.5 recalls per girl). Among girls with more than one recall, the mean number of recalls was 3.2 and the median range of SSB intake was 383 ml/day across recalls.

### Covariates

Total dairy intake was assessed as the total of any food or beverages derived from milk products, including milk, cheese, and cream but excluding milk-based beverages with added sugar. Total meat intake was the totality of all meat from beef, chicken, pork, and turkey, in any form. Anthropometric assessments were performed every 6–12 months during follow-up to measure weight and height. Menarche (yes/no), which typically occurs during Tanner stage 4 or 5, was self-reported during brief phone interviews that occurred every 3 months with trained dietitians [37]. Body mass index (BMI; kg/m^2^) was calculated as weight divided by height squared and then age- and sex-standardized in accordance with WHO Child Growth Reference (2007) [38]. During the clinic visit, mothers of the children self-reported their highest education, and their weight and height were measured. Data on the number of hours of television watched by the children after school was also collected via self-report as a proxy for physical activity.

### Statistical analysis

Baseline demographics are presented as mean (SD) if continuous or as a frequency (percentage) if categorical. To account for non-linear associations, SSB intake was modeled using quartiles. Linear regression models were used to evaluate the association between SSB intake quartiles and continuous breast outcomes (aFGV, %FGV, and tBV). Two multivariable linear regression models were evaluated to account for potential confounders. Model 1 was adjusted for BMI Z-score, age at Tanner 4, and daily energy intake (total kCal/day). Model 2 was adjusted for model 1 plus maternal education level, hours of daily television watching after school, dairy intake (g/day), meat intake (g/day), waist circumference, and menarche. To evaluate the sensitivity of the potential association to the number of recalls for each girl and to account for potential issues of temporality, we additionally stratified the analysis by number of 24H dietary recalls (one recall and > one recall) and evaluated the associations between SSB intake and continuous breast outcomes. Effect estimates presented represent absolute differences in outcomes in the second, third, and fourth quartiles of intake compared to the first (the reference category). Significance trends were evaluated by modeling the median SSB intake within quartiles as a continuous variable.

Body composition is a strong influencer of breast density measurements and a known associate of diet. Therefore, to characterize the relation of body composition to breast outcomes and SSB intake in our cohort, we used linear regression models to evaluate the strength of the association between SSB intake quartiles and anthropometric measures (BMI Z-score, body fat percentage, and waist circumference) as a supplemental analysis. Model 1 was adjusted for daily energy level (total kCal/day). Model 2 was adjusted for model 1 plus age at visit, dairy intake (g/day), and meat intake (g/day). Model 3 was adjusted for model 2 plus maternal education level and hours of daily television watching after school. We further examined the association between measures of body composition and breast composition at Tanner stage 4 in our cohort.

To evaluate whether our primary results were influenced by Tanner stage, we subset our sample to include only girls whose breast assessments were done at Tanner stage 4, excluding the 22% of the cohort with a breast assessment at Tanner stage 5, and ran the same linear regression models. We also performed simple sensitivity analyses to evaluate whether any potential SSB intake and breast outcome relation were sensitive to misreporting of SSB intake by girls with the highest BMI. To account for potential underreporting of SSB intake, we divided continuous (ml/day) SSB intake by 0.95 and 0.80 among obese girls (BMI Z-score ≥ 2), cut intake into quartiles, then carried out standard linear regression analyses as described above.

All statistical tests were 2-sided and were performed at the 0.05 level of significance. We used SAS 9.4 software (SAS Institute Inc.) for all analyses.

## Results

**Fig. 1:**
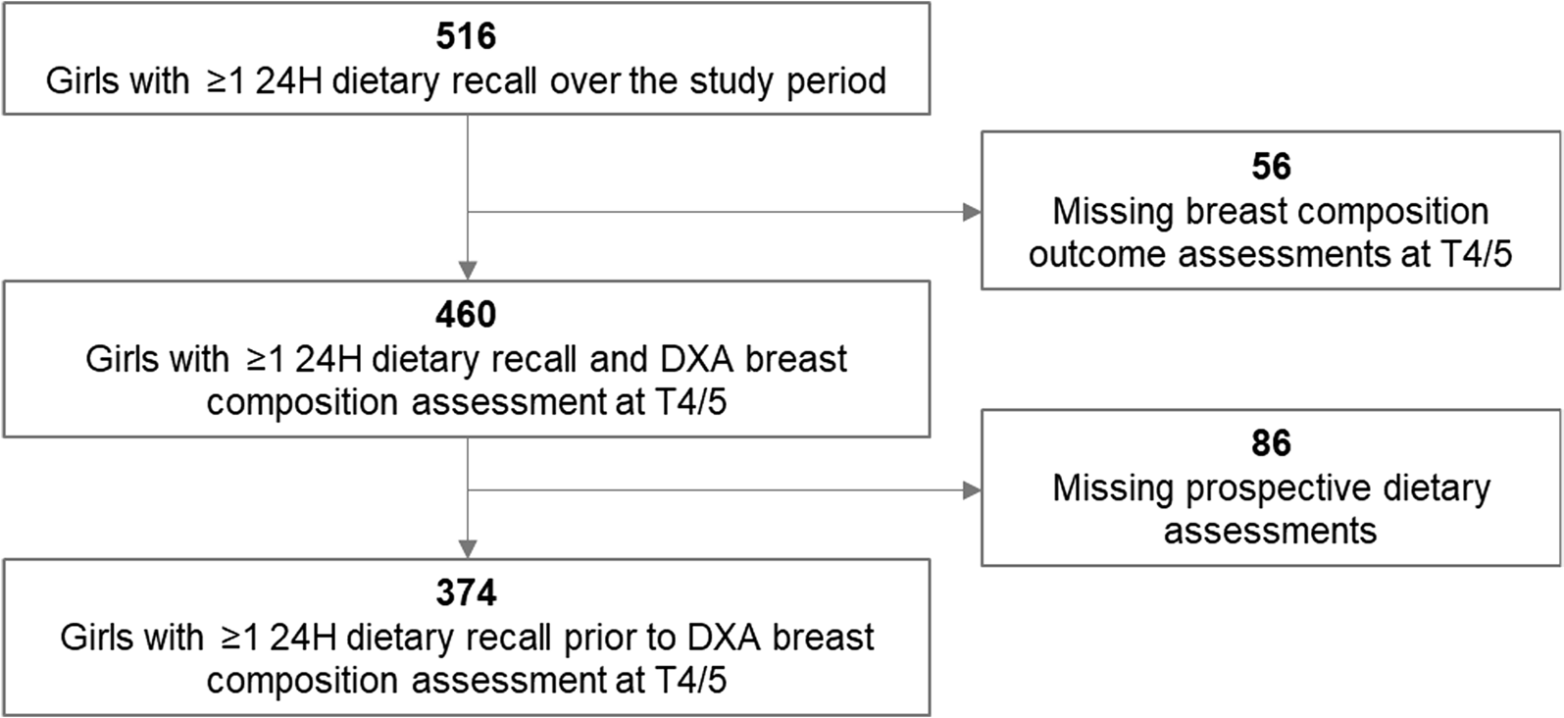
Overview of participant exclusion for SSB intake and breast density analyses in GOCS

This study includes 374 girls participating in GOCS assessed for breast development at Tanner stage 4 or 5 with at least one prospective 24H dietary recall (Fig. 1). There were no important differences in characteristics of the 374 girls included in this analysis and the 515 girls in the larger GOCS cohort (Additional file 1: Table S1). The mean age at the Tanner 4 clinic visit was 11.5 years (SD 0.8) (Table 1). Of the 374 girls in the cohort, 61.8% had more than one prospective 24H recall; 38.2% had only one. Among the girls with only one 24H dietary recall, mean follow-up time was < one month, while girls with more than one 24H recall had mean follow-up of 7.9 months (data not shown). Overall, the mean ± SD daily SSB intake was 342 ± 210 mL, which is roughly equivalent to one 12-oz soda can (355 mL). Mean SSB intake for each quartile was 100 mL, 253 mL, 376 mL, and 626 mL per day. On average, girls in the second quartile (Q2) of SSB intake had slightly high BMIs than girls in the first, third, and fourth quartiles (BMI Z-score 1.0 in Q2, compared to 0.9, 0.7, and 0.6 in Q1, Q3, and Q4, respectively); this trend was consistent for other anthropometric characteristics including total fat percentage (fat %) and waist circumference. Overall, the mean ± SD breast FGV, %FGV, and tBV at Tanner 4 in the cohort was 86.5 ± 35.2 cm^3^, 41.7% ± 16.5%, and 232.2 ± 114.4 cm^3^, respectively. Other study characteristics are presented in Table 1.

**Table 1:** Characteristics of girls in the Growth and Obesity Cohort Study (N = 374)

Characteristic	Total		Quartile of sugar-sweetened beverage (SSB) intake							
			Q1 (n = 88)		Q2 (n = 99)		Q3 (n = 92)		Q4 (n = 95)	
	Mean	SD	Mean	SD	Mean	SD	Mean	SD	Mean	SD
SSB intake, ml/day (mean, range)	342.1	210.0	100.0	(0, 193.8)	253.3	(200.0, 315.6)	375.7	(315.8, 446.4)	626.2	(450.0, 1143.3)
Age at Tanner 4 visit, years	11.5	0.8	11.2	0.7	11.6	0.8	11.7	0.9	11.6	0.8
*DXA breast characteristics*										
Absolute fibroglandular volume, cm^3^	86.5	35.2	84.1	39.7	83.4	33.5	88.6	32.4	89.8	35.1
% Fibroglandular volume	41.7	16.5	40.7	18.1	38.2	15.4	42.9	15.9	45.3	16.0
Total breast volume, cm^3^	232.2	114.4	236.1	123.5	246.0	127.6	227.7	98.6	218.7	104.9
*Menarche (n, %)*										
No	311	84	73	84	83	84	78	85	77	82
Yes	61	16	14	16	16	16	14	16	17	15
*Anthropometric characteristics*										
BMI Z-score	0.8	1.1	0.9	1.1	1.0	1.1	0.7	1.2	0.6	1.1
Fat %	26.8	5.3	26.9	6.0	28.3	5.2	26.6	4.9	25.6	4.7
Waist circumference (cm)	72.5	9.6	72.7	10.4	74.6	9.8	72.2	8.5	70.4	9.5
*Hours of TV per day (n, %)*										
0–2 h	26	8	6	8	7	8	3	4	10	12
2–4 h	119	38	33	45	20	24	35	47	31	38
> 4 h	170	54	35	47	57	68	37	49	41	50
*Number of 24-h recalls (n, %)*										
1	143	38	50	57	35	35	25	27	33	35
2	75	20	21	24	13	13	19	21	22	23
3	74	20	12	14	20	20	18	20	24	25
4+ recalls	82	22	5	6	31	31	30	33	16	17
*Dietary intake (per day)*										
Total calories, kCal	1874.4	546.0	1783.2	642.6	1761.0	476.9	1876.5	431.1	2075.2	567.1
From fat, %	29.9	6.2	30.1	7.8	29.1	6.5	30.4	5.5	30.1	4.8
From protein, %	13.9	3.0	15.2	3.6	14.0	2.5	13.7	3.1	12.7	2.5
From carbohydrates, %	57.5	6.6	55.9	8.1	58.3	6.4	57.2	6.2	58.6	5.3
Meat, g/day	85.9	56.4	87.9	64.2	82.7	52.0	87.9	57.9	85.4	52.1
Dairy, g/day	226.4	160.0	225.6	188.8	215.4	159.1	236.1	151.2	229.2	140.7
*Maternal characteristics*										
BMI (kg/m^2^)	27.1	5.4	27.0	5.6	27.6	5.0	26.8	5.8	27.0	5.1
*Highest education level (n, %)*										
Less than high school	70	19	12	14	22	23	14	17	22	25
High school	225	60	60	71	59	62	56	67	50	56
University	57	15	13	15	14	15	13	16	17	19

Categorical variables may not sum to 374 due to missing values

We found no evidence to suggest an association between SSB intake and aFGV, %FGV, or tBV (Table 2). On average, aFGV was not different for girls in SSB intake Q2 (β: − 5.4, 95% CI − 15.1, 4.4), Q3 (β: 1.3, 95% CI − 8.6, 11.3), and Q4 (β: 3.0, 95% CI − 7.1, 13) compared to girls in SSB intake Q1 (found our data to be compatible with the null hypothesis (*p-*trend = 0.29). The addition of maternal education level, hours of daily television watching after school, dairy intake (g/day), meat intake (g/day), waist circumference, and menarche as potential confounders did not alter the association between SSB intake and aFGV (Model 2; Table 2). Similar null associations were seen for the relation between SSB intake and %FGV and tBV.

**Table 2:** Association between sugar-sweetened beverage intake and breast composition at Tanner 4 among GOCS girls (N = 374)

SSB Quartile	n	Model 1		Model 2	
		β	95% CI	β	95% CI
*Absolute fibroglandular volume (cm*^*3*^*)*					
Q1	88	Ref		Ref	
Q2	92	− 5.4	(− 15.1, 4.4)	− 4.5	(− 15.2, 6.3)
Q3	99	1.3	(− 8.6, 11.3)	0.0	(− 11.0, 11.1)
Q4	95	3	(− 7.1, 13)	1.5	(− 9.5, 12.5)
p-trend		0.29		0.66	
*Percent fibroglandular volume (%)*					
Q1	88	Ref		Ref	
Q2	92	− 1.1	(− 4.4, 2.1)	− 0.6	(− 4.0, 2.8)
Q3	99	0.5	(− 2.9, 3.8)	1.6	(− 1.9, 5.1)
Q4	95	1.3	(− 2, 4.6)	0.8	(− 2.1, 4.9)
p-trend		0.26		0.28	
*Total volume (cm*^*3*^*)*					
Q1	88	Ref		Ref	
Q2	92	− 11.6	(− 33.3, 10.2)	− 13.2	(− 34.9, 8.5)
Q3	99	− 8.5	(− 30.8, 13.7)	− 17.0	(− 39.4, 5.4)
Q4	95	− 7.0	(− 29.4, 15.4)	− 9.0	(− 31.2, 13.2)
p-trend		0.71		0.57	

All β estimates and 95% confidence intervals are obtained from linear regression models. Confidence intervals that do not include 0 are in bold. Model 1 was adjusted for BMI Z-score, age at Tanner stage 4, and daily energy level (total kCal/day). Model 2 was adjusted for model 1 plus maternal education level, hours of daily television watching after school, dairy intake (g/day), meat intake (g/day), waist circumference, and menarche. βs represent absolute differences in outcomes in the second, third, or fourth quartile of SSB intake compared to the first (the reference category). Significant trends were evaluated by modeling the median SSB intake within quartiles as a continuous variable and presented as *p* values

*SSB* sugar-sweetened beverage

These null results were robust to stratification by number of 24H recalls. Among girls with more than one 24H dietary recall, we found no associations with aFGV, %FGV, and tBV for girls in SSB Q2, Q3, and Q4 compared to Q1 (Table 3). The lack of association was not modified when adjusting for additional potential confounders. Null associations were also seen among girls with only one 24H recall for SSB intake and aFGV and tBV. However, we did find that among girls with one 24H recall, those in SSB intake Q2 had modestly lower %FGV compared to those in SSB intake Q1 (Model 1 β: − 6.1, 95% CI − 10.7, − 1.5) when adjusting for potential confounders (*P*-trend = 0.28).

**Table 3:** Stratified association between SSB intake and breast composition at Tanner 4 among GOCS girls (N = 374)

SSB quartile	Girls with one 24H recall				Girls with > one 24H recall			
	Model 1		Model 2		Model 1		Model 2	
	β	95% CI	β	95% CI	β	95% CI	β	95% CI
	Absolute fibroglandular volume (cm^3^)				Absolute fibroglandular volume (cm^3^)			
Q1	Ref		Ref		Ref		Ref	
Q2	− 10.8	(− 24.9, .2)	− 12.7	(− 28.3, 2.9)	− 4.1	(− 17, 8.8)	− 6.2	(− 20.6, 8.2)
Q3	− 7.3	(− 20.8, 6.2)	− 9.6	(− 24.1, 4.9)	− 2.8	(− 15.8,10.2)	− 6.7	(− 21.7, 8.2)
Q4	5.9	(− 7.7, 19.6)	3.2	(− 11.6, 18.1)	− 0.5	(− 13.8,12.8)	− 5.0	(− 19.8, 9.7)
p-trend	0.24		0.39		0.92		0.62	

	Percent fibroglandular volume (%)				Percent fibroglandular volume (%)			
Q1	Ref		Ref		Ref		Ref	
Q2	− 6.1	**(**− **10.7, **− **1.5)**	− 6.7	**(**− **11.8, **− **1.5)**	0.5	(− 3.9, 4.8)	0.1	(− 4.4, 4.6)
Q3	− 2.1	(− 6.5, 2.3)	− 2.2	(− 7, 2.6)	− 1.1	(− 3.9, 3.3)	− 1.2	(− 5.9, 3.5)
Q4	1.0	(− 3.5, 5.4)	0.6	(− 4.3, 5.6)	− 0.5	(− 4.9, 4.0)	− 0.4	(− 5, 4.2)
p-trend	0.28	0.30	0.75	0.81				

	Total volume (cm^3^)				Total volume (cm^3^)			
Q1	Ref		Ref		Ref		Ref	
Q2	− 2.7	(− 33.4, 27.9)	− 3.2	(− 35.7, 29.4)	− 15.8	(− 44.8, 13.3)	− 17.9	(− 46.5, 10.7)
Q3	− 25.2	(− 54.8, 4.3)	− 27.7	(− 58.1, 2.7)	− 6.5	(− 35.8, 22.8)	− 14.8	(− 44.5, 14.9)
Q4	− 1.3	(− 31.1, 28.4)	− 0.4	(− 31.5, 30.7)	− 9.6	(− 39.6, 20.3)	− 14.1	(− 43.3, 15.2)
p-trend	0.77		0.87		0.71		0.50	

All β estimates and 95% confidence intervals are obtained from linear regression models. Confidence intervals that do not include 0 are in bold. Model 1 was adjusted for BMI Z-score, age at Tanner stage 4, and daily energy level (total kCal/day). Model 2 was adjusted for model 1 plus maternal education level, hours of daily television watching after school, dairy intake (g/day), meat intake (g/day), waist circumference, and menarche. βs represent absolute differences in outcomes in the second, third, or fourth quartile of SSB intake compared to the first (the reference category). Significant trends were evaluated by modeling the median SSB intake within quartiles as a continuous variable and presented as *p* values

*SSB* sugar-sweetened beverage

In supplementary analyses, we found no relation between SSB intake quartile and measures of anthropometry (Additional file 1: Table S2). There was no difference in BMI Z-score, body fat percentage, or waist circumference for girls in SSB intake quartiles Q2–Q4 compared to Q1. The association remained null when adjusting for different sets of potential confounders. However, measures of anthropometry were associated with breast outcomes. BMI Z-score was inversely correlated with %FGV (ρ = − 0.73) and positively correlated with tBV (ρ = 0.70) (data not shown). In multivariable linear models, BMI Z-score was associated with higher aFGV (β = 5.6, 95% CI 2.4, 8.9), lower %FGV (β = − 11.0, 95% CI − 12.1, − 9.9), and higher tBV (β = 79.4, 95% CI 72.1, 86.6) (Additional file 1: Table S3). Waist circumference was also positively associated aFGV and tBV, and inversely associated with %FGV.

In sensitivity analyses, which excluded girls with breast assessments at Tanner stage 5, SSB intake was not associated with aFGV, %FGV, or tBV (Additional file 1: Table S4). Though we did note suggestions of a shift in the positive direction for the association of SSB intake to aFGV in Model 2 when comparing the results of this analysis to those from Table 2, the effect estimates were not statistically significant. To account for potential misreporting among girls with higher body size, we re-analyzed the association between SSB intake and breast outcomes under assumption that’s obese girls might underreport their SSB intake by 5% of 20% (Additional file 1: Table S5). In these sensitivity analyses, we found no association between SSB intake and any breast outcome under this set of assumptions.

## Discussion

To our knowledge, this is the first study to evaluate the association between intake of sugar-sweetened beverages and breast density in a cohort of adolescent girls. Overall, we observed no associations between SSB intake and breast outcomes, including breast density and breast volume. This null relation remained consistent after adjustment for potential confounders such as maternal education and when accounting for potential measurement error of SSB intake.

The lack of associations between SSB intake and measures of breast density among a subset of participants with more than one 24H recall suggests that our results may be robust to a true null relation. To our knowledge, only one other population-based study has examined the relation of sugary beverages to breast density [10]. A cross-sectional study of 1555 pre- and post-menopausal Canadian women with no history of breast cancer found that SSB intake was positively associated with mammographic density when comparing those in the highest category to those in the lowest category of SSB intake [10]. In contrast with our cohort, the study used food frequency questionnaires to evaluate SSB intake, mammographic density rather than DEXA, and the average age of women in the study was 54 years. While no other studies have examined breast density as an outcome, SSB intake has been positively associated with breast cancer risk and mortality across population-based studies and in meta-analyses [39–41]. The inconsistent relations of SSB intake to breast density and breast cancer may reflect the complex relation to body fatness. SSB consumption has been positively associated with obesity and weight gain across the lifespan [42]. There is also considerable evidence that increased body fatness in early adulthood is inversely associated with risk of later-life breast cancer [43, 44]. Moreover, several studies report strong associations between body fatness and breast composition in girls and women across the life course, including pre- and post-menopause [42, 45–47]. The results from our study support a similar relation between pubertal body composition and adolescent breast density. Specifically, we report consistency with published literature for the association between body composition and breast composition: BMI Z-score, body fat percentage, and waist circumference are positively associated with tBV and negatively associated with %FGV. However, we did not find a relation between SSB intake and body composition our cohort, which may explain the null association we observed between SSB intake and breast density.

It is possible that the null associations in our study may be partially explained by misclassification of SSB intake. Underreporting is a major limitation in the collection of self-reported dietary intake; obese individuals, both adult and adolescent, are more likely to underreport total energy intake [48–51]. Furthermore, intake of sodas and other high-calorie foods and beverages is more likely to be underestimated by study participants in general [52, 53]. Results from prospective cohort studies support a positive and consistent association between SSB intake and body mass or fatness among children [42, 54]. In contrast, we did not observe differences in body composition metrics (BMI Z-score, fat %, waist circumference) for girls in our cohort reporting higher levels of SSB intake compared to those with lower SSB intake. This is surprising, particularly given that girls in the highest SSB intake quartile report the highest total caloric intake. Moreover, we also report no association between SSB intake and total breast volume, a measure of breast size that is closely correlated with body size. Taken together, these observations suggest there may be differential underreporting of SSB intake by body size. However, in sensitivity analyses that assume obese girls may underreport SSB intake by as much as 20%, the associations between SSB intake and breast outcomes were also null. While these assumptions suggest minimal influence of misclassification on our results, the extent to which potential differential misclassification of SSB intake contributed to the observed trends cannot be fully assessed. Current methods to quantify underreporting in self-reported dietary methods are limited to estimating true total energy intake [55–57]. For example, underreporting of energy intake has been observed to range from 5 to 25% in studies conducted among Latin American and European adolescents [58, 59]. However, there is little knowledge about the distribution of such measurement error specific to individual foods (e.g., SSB) or dietary nutrients (e.g., sugar) in these populations, particularly across different anthropometric characteristics of body composition.

We had previously proposed that a positive association between sweetened artificially-flavored milk-based drinks and breast %FGV in the GOCS cohort was driven by the sugar content of the drinks [27]. In the current analysis, we attempted to test this hypothesis by expanding the type of sugary beverages evaluated to include popularly consumed products such as carbonated sodas, sweetened coffees, and fruit juices with added sugar. Altogether, these beverages are in the most frequently consumed by volume among Chilean adolescents and represent almost 15% of total daily caloric intake, corresponding to approximately 235 kCal from SSB [35]. That we fail to observe the same positive association in our analyses might suggest conflation of different sources of sugar consumption. We lacked the ability to distinguish between sugars such as sucrose or fructose that may have differential impacts on body development. For instance, a United States-based study found that adolescent sucrose intake, but not fructose, was positively associated with dense breast volume in early adulthood [60]. Sucrose, a common table sugar, is also associated with increased BMI among adults [61]. Evidence for the effect of fructose, which is commonly added to SSBs in the form of high-fructose corn syrup, on obesity and other health outcomes is mixed [62]. Additionally, we also lacked information on ultra-processed foods (UPFs), which are positively associated with body composition and have been linked to breast cancer outcomes [23, 63, 64]. Recently, a large ongoing study of Latin American countries recently found that higher intake of UPFs was associated with increased breast cancer risk [65]. A broader assessment of UPFs in our cohort would be needed to explore this hypothesis.

Several limitations of this study exist. Self-report of diet is subject to measurement error and may misrepresent the habitual dietary intake. However, most of the girls had more than one 24H recall, limiting the effect of random within-person variation and allowing for a better representation of average diet. Of course, this does not preclude the likelihood of systematic underreporting of specific dietary components such as SSBs. Because this is an observational study, we also cannot rule out the possibility of confounding by unmeasured lifestyle, dietary, or genetic factors. Another limitation of this study is the relatively homogenous racial/ethnic makeup of the cohort, limiting generalizability to other populations.

Major strengths of the study include its prospective design and repeated assessments of diet. In addition, we have detailed anthropometric information and the ability to evaluate several different measures of body composition as potential confounding variables. We were also able to control for time spent watching television as a proxy for physical activity, an important confounder of the association between SSB and obesity among adolescents and therefore a proposed confounder of the SSB and breast density association [66]. Finally, this study is among the few to evaluate the relation between diet and breast composition during adolescence, a critical period of breast development and an important window of susceptibility in the life course conceptualization of breast cancer risk. Prior studies have suggested that breast density is most variable in girls and younger women and eventually attains a lower and more consistent value as the population ages [67].

## Conclusions

In summary, our study does not provide evidence for an association between breast composition and SSB intake during adolescence. However, measurement error may mask a true relation.

## Supplementary Information

**Additional file 1. Supplemental Table 1.** Comparison of key sample characteristics for girls in the Growth and Obesity Cohort Study (N=515) and those included in the analytic cohort for the sugar-sweetened beverage and breast outcome analysis at Tanner Stage 4 (N=374); **Supplemental Table 2**. Association between SSB intake quartile and anthropometric measures of body composition at Tanner stage 4 among 374 girls in the Growth and Obesity Cohort Study;  **Supplemental Table 3**. Association between body composition characteristics and breast composition at Tanner stage 4 among 374 girls in the Growth and Obesity Cohort Study;  **Supplemental Table 4**. Association between sugar-sweetened beverage (SSB) intake and breast composition at Tanner stage 4 among 293 girls in the Growth and Obesity Cohort Study assessed between 2013-2016 in Santiago, Chile, excluding girls with breast assessments at Tanner stage 5;  **Supplemental Table 5**. Association between sugar-sweetened beverage (SSB) intake and breast composition at Tanner stage 4 among 374 girls in the Growth and Obesity Cohort Study assessed between 2013-2016 in Santiago, Chile assuming underreporting of SSB by 5% and 20% among obese girls

## Data Availability

The datasets used and/or analyzed during the current study are available from the corresponding author on reasonable request.

## Abbreviations

SSB: Sugar-sweetened beverages
IGF-1: Insulin-like growth factor
UPF: Ultra-processed foods
MRI: Magnetic resonance imaging
DXA: Dual-energy X-ray absorptiometry
GOCS: Growth and Obesity Cohort Study
JUNJI: National Association of Dare Care Centers
FGV: Fibroglandular volume
aFGV: Absolute fibroglandular volume
%FGV: Percent fibroglandular volume
tBV: Total breast volume
BMI: Body mass index
SD: Standard deviation
CI: Confidence interval
Q[1–4]: Quartile[1–4]
