# Supplementary material for: Sugar-sweetened beverage consumption and breast composition in a longitudinal study of Chilean girls
Source: Breast Cancer Res. 2022 Jan 8;24:3. doi: 10.1186/s13058-021-01495-8 (PMC8742361; doi:10.1186/s13058-021-01495-8)
Supplement: Supplementary file 1 — Additional file 1. Supplemental Table 1. Comparison of key sample characteristics for girls in the Growth and Obesity Cohort Study (N=515) and those included in the analytic cohort for the sugar-sweetened beverage and breast outcome analysis at Tanner Stage 4 (N=374); Supplemental Table 2. Association between SSB intake quartile and anthropometric measures of body composition at Tanner stage 4 among 374 girls in the Growth and Obesity Cohort Study; Supplemental Table 3. Association between body composition characteristics and breast composition at Tanner stage 4 among 374 girls in the Growth and Obesity Cohort Study; Supplemental Table 4. Association between sugar-sweetened beverage (SSB) intake and breast composition at Tanner stage 4 among 293 girls in the Growth and Obesity Cohort Study assessed between 2013-2016 in Santiago, Chile, excluding girls with breast assessments at Tanner stage 5; Supplemental Table 5. Association between sugar-sweetened beverage (SSB) intake and breast composition at Tanner stage 4 among 374 girls in the Growth and Obesity Cohort Study assessed between 2013-2016 in Santiago, Chile assuming underreporting of SSB by 5% and 20% among obese girls [file 13058_2021_1495_MOESM1_ESM.docx]

**Supplemental Table 1.** Comparison of key sample characteristics for girls in the Growth and Obesity Cohort Study (N=515) and those included in the analytic cohort for the sugar-sweetened beverage and breast outcome analysis at Tanner Stage 4 (N=374)

|  | **Full cohort (N=515)** | | **Analytic cohort (N=374)** | |
| --- | --- | --- | --- | --- |
| **Characteristic** | **Total** | | **Total** | |
|  | **Mean** | **SD** | **Mean** | **SD** |
| Age at Tanner Stage 4 or 5 visit, years | 11.3 | 0.9 | 11.5 | 0.8 |
| Menarche (n,%) |  |  |  |  |
| No | 387 | 75.1% | 311 | 83.2% |
| Yes | 71 | 13.8% | 61 | 16.3% |
| Missing | 57 | 11.1% | 2 | 0.5% |
| Anthropometric characteristics |  |  |  |  |
| BMI-Z score | 0.9 | 1.1 | 0.8 | 1.1 |
| Fat % | 26.9 | 5.2 | 26.8 | 5.3 |
| Waist circumference | 72.5 | 9.3 | 72.5 | 9.6 |
| Hours of TV per day (n,%) |  |  |  |  |
| 0 - 2 hours | 32 | 6.2% | 26 | 7.0% |
| 2 - 4 hours | 150 | 29.1% | 119 | 31.8% |
| >4 hours | 206 | 40.0% | 170 | 45.5% |
| Missing | 127 | 24.7% | 59 | 15.8% |
| Mother's characteristics |  |  |  |  |
| BMI (kg/m2) | 27.1 | 5.3 | 27.1 | 5.4 |
| Highest education level (n,%) |  |  |  |  |
| Less than high school | 58 | 11.2% | 70 | 18.7% |
| High school | 225 | 43.6% | 225 | 60.2% |
| University | 72 | 14.0% | 57 | 15.2% |
| Missing | 161 | 31.2% | 22 | 5.9% |

**Supplemental Table 2**. Association between SSB intake quartile and anthropometric measures of body composition at Tanner stage 4 among 374 girls in the Growth and Obesity Cohort Study

|  | **Model 1** | |  | **Model 2** | |  | **Model 3** | |  |
| --- | --- | --- | --- | --- | --- | --- | --- | --- | --- |
| **SSB Quartile** | **β** | **95% CI** |  | **β** | **95% CI** |  | **β** | **95% CI** |  |
| BMI Z-score | | | | | | | | |  |
| Q1 | ref | |  | ref | |  | ref | |  |
| Q2 | 0.07 | (-0.24, 0.38) |  | 0.20 | (-0.1, 0.5) |  | 0.16 | (-0.17, 0.49) |  |
| Q3 | -0.16 | (-0.48, 0.16) |  | -0.01 | (-0.32, 0.31) |  | 0.09 | (-0.25, 0.43) |  |
| Q4 | -0.22 | (-0.54, 0.1) |  | -0.07 | (-0.39, 0.24) |  | 0.01 | (-0.32, 0.35) |  |
| p-trend | 0.08 | |  | 0.32 | |  | 0.82 | |  |
| Body fat percentage | | | | | | | | |  |
| Q1 | ref | |  | ref | |  | ref | |  |
| Q2 | 1.34 | (-0.12, 2.8) |  | 1.42 | (-0.06, 2.9) |  | 1.19 | (-0.41, 2.8) |  |
| Q3 | -0.16 | (-1.65, 1.33) |  | -0.05 | (-1.56, 1.47) |  | 0.45 | (-1.22, 2.12) |  |
| Q4 | -0.82 | (-2.32, 0.69) |  | -0.72 | (-2.25, 0.81) |  | -0.21 | (-1.86, 1.43) |  |
| p-trend | 0.07 | |  | 0.09 | |  | 0.44 | |  |
| Waist circumference | | | | | | | | |  |
| Q1 | ref | |  | ref | |  | ref | |  |
| Q2 | 1.73 | (-0.95, 4.4) |  | 1.74 | (-0.97, 4.44) |  | 1.45 | (-1.51, 4.4) |  |
| Q3 | -0.23 | (-2.96, 2.5) |  | -0.20 | (-2.98, 2.57) |  | 0.85 | (-2.22, 3.93) |  |
| Q4 | -1.31 | (-4.06, 1.44) |  | -1.28 | (-4.08, 1.51) |  | -0.23 | (-3.26, 2.81) |  |
| p-trend | 0.14 | |  | 0.14 | |  | 0.69 | |  |
| Note: All β estimates and 95% confidence intervals are obtained from linear regression models. Confidence intervals that do not include 0 are in **bold**. Model 1 was adjusted for daily energy level (total kCal/day). Model 2 was adjusted for model 1 plus age at visit, dairy intake (g/day) and meat intake (g/day). Model 3 was adjusted for model 2 plus maternal education level and hours of daily television watching after school. βs represent absolute differences in outcomes in the second, third, or fourth quartile of SSB intake compared to the first (the reference category). Significant trends were evaluated by modeling the median SSB intake within quartiles as a continuous variable and presented as p-values. | | | | | | | | |  |
|  |  |  |  |  |  |  |  |  |  |
|  |  |  |  |  |  |  |  |  |  |

**Supplemental Table 3.** Association between body composition characteristics and breast composition at Tanner stage 4 among 374 girls in the Growth and Obesity Cohort Study

|  |  | **Model 1** | |  | **Model 2** | |
| --- | --- | --- | --- | --- | --- | --- |
|  |  | β | 95% CI |  | β | 95% CI |
| Absolute fibroglandular volume (cm^3^) | | | | | | |
| BMI Z-score |  | 3.1 | (-0.1,6.2) |  | 5.6 | **(2.4,8.9)** |
| Fat percentage |  | 0.5 | (-0.2,1.2) |  | 0.6 | (-0.1,1.2) |
| Waist circumference |  | 0.6 | **(0.2,0.9)** |  | 0.6 | **(0.2,0.9)** |
| Percent fibroglandular volume (%) | | | | | | |
| BMI Z-score |  | -10.8 | **(-11.8,-9.8)** |  | -11.0 | **(-12.1,-9.9)** |
| Fat percentage |  | -2.4 | **(-2.6,-2.2)** |  | -2.4 | **(-2.6,-2.2)** |
| Waist circumference |  | -1.2 | **(-1.3,-1.1)** |  | -1.2 | **(-1.3,-1.1)** |
| Total breast volume (cm^3^) | | | | | | |
| BMI Z-score |  | 70.9 | **(63.5,78.4)** |  | 79.4 | **(72.1,86.6)** |
| Fat percentage |  | 16.1 | **(14.6,17.5)** |  | 16.0 | **(14.6,17.5)** |
| Waist circumference |  | 9.1 | **(8.3,9.9)** |  | 9.1 | **(8.3,9.9)** |
| Note: All β estimates and 95% confidence intervals are obtained from linear regression models. Confidence intervals that do not include 0 are in **bold**. Model 1 was unadjusted. Model 2 was adjusted for age and daily energy intake. | | | | | | |

**Supplemental Table 4.** Association between sugar-sweetened beverage (SSB) intake and breast composition at Tanner stage 4 among 293 girls in the Growth and Obesity Cohort Study assessed between 2013-2016 in Santiago, Chile, excluding girls with breast assessments at Tanner stage 5

|  |  | **Model 1** | | **Model 2** | |
| --- | --- | --- | --- | --- | --- |
| **SSB Quartile** | **n** | β | 95% CI | β | 95% CI |
| *Absolute fibroglandular volume (cm^3^)* | | | | | |
| Q1 | 74 | ref | | ref | |
| Q2 | 80 | 0.0 | (-9.8,9.8) | 1.6 | (-9.2,12.4) |
| Q3 | 69 | 4.2 | (-6,14.4) | 3.3 | (-8.1,14.7) |
| Q4 | 70 | 5.8 | (-4.6,16.2) | 6.7 | (-4.6,17.9) |
| p-trend |  | 0.20 | | 0.22 | |
| *Percent fibroglandular volume* | | | | | |
| Q1 | 74 | ref | | ref | |
| Q2 | 80 | 0.2 | (-3.3,3.7) | 0.2 | (-3.4,3.9) |
| Q3 | 69 | 0.9 | (-2.7,4.6) | 1.5 | (-2.3,5.4) |
| Q4 | 70 | 1.4 | (-2.3,5.1) | 1.2 | (-2.6,5) |
| p-trend |  | 0.41 | | 0.47 | |
| *Total volume (cm^3^)* | | | | | |
| Q1 | 74 | ref | | ref | |
| Q2 | 80 | -12.1 | (-32.8,8.7) | -9.4 | (-30.2,11.4) |
| Q3 | 69 | -4.8 | (-26.3,16.7) | -9.3 | (-31.4,12.7) |
| Q4 | 70 | -1.0 | (-22.9,21) | 1.8 | (-19.9,23.6) |
| p-trend |  | 0.82 | | 0.70 | |
| Note: All β estimates and 95% confidence intervals are obtained from linear regression models. Confidence intervals that do not include 0 are in **bold**. Model 1 was adjusted for BMI Z-score, age at Tanner stage 4, and daily energy level (total kCal/day), and menarche. Model 2 was adjusted for model 1 plus maternal education level, hours of daily television watching after school, dairy intake (g/day), meat intake (g/day), and waist circumference. Significant trends were evaluated by modeling the median SSB intake within quartiles as a continuous variable and presented as p-values. | | | | | |

**Supplemental Table 5.** Association between sugar-sweetened beverage (SSB) intake and breast composition at Tanner stage 4 among 374 girls in the Growth and Obesity Cohort Study assessed between 2013-2016 in Santiago, Chile assuming underreporting of SSB by 5% and 20% among obese girls

|  | **SSB underreporting of 5% among obese girls** | | | | **SSB underreporting of 20% among obese girls** | | | |
| --- | --- | --- | --- | --- | --- | --- | --- | --- |
|  | **Model 1** |  | **Model 2** |  | **Model 1** |  | **Model 2** |  |
| **SSB Quartile** | **β** | **95% CI** | **β** | **95% CI** | **β** | **95% CI** | **β** | **95% CI** |
|  | *Absolute fibroglandular volume (cm^3^)* | | | | *Absolute fibroglandular volume (cm^3^)* | | | |
| Q1 | ref |  | ref |  | ref |  | ref |  |
| Q2 | -2.2 | (-12.1,7.7) | -1.7 | (-12.7,9.3) | 0.1 | (-9.8,10) | 0.4 | (-10.7,11.5) |
| Q3 | 4.4 | (-5.4,14.1) | 1.4 | (-9.7,12.6) | 4.8 | (-5.1,14.7) | 1.9 | (-9.3,13.1) |
| Q4 | 4.1 | (-5.8,14.1) | 1.7 | (-9.3,12.8) | 4.1 | (-5.8,14.1) | 2.2 | (-9,13.3) |
| p-trend | 0.27 | | 0.56 | | 0.41 | | 0.61 | |
|  | *Percent fibroglandular volume (%)* | | | | *Percent fibroglandular volume (%)* | | | |
| Q1 | ref |  | ref |  | ref |  | ref |  |
| Q2 | 0 | (-3.3,3.3) | 0 | (-3.4,3.5) | -0.5 | (-3.7,2.8) | 0 | (-3.5,3.5) |
| Q3 | 1.5 | (-1.8,4.7) | 1.7 | (-1.8,5.2) | 1.4 | (-1.8,4.7) | 1.5 | (-2.0,5.0) |
| Q4 | 1.7 | (-1.6,5) | 1.9 | (-1.5,5.4) | 1.7 | (-1.6,5) | 2.1 | (-1.4,5.7) |
| p-trend | 0.24 | | 0.18 | | 0.25 | | 0.15 | |
|  | *Total breast volume (cm^3^)* | | | | *Total breast volume (cm^3^)* | | | |
| Q1 | ref |  | ref |  | ref |  | ref |  |
| Q2 | -9 | (-31.1,13.0) | -8 | (-30.3,14.2) | 3.3 | (-18.8,25.3) | 1 | (-21.4,23.4) |
| Q3 | -1.2 | (-23,20.5) | -9.9 | (-32.3,12.6) | 0.4 | (-21.6,22.4) | -8.7 | (-31.3,13.9) |
| Q4 | -5.6 | (-27.7,16.6) | -9.9 | (-32.2,12.4) | -5.5 | (-27.7,16.6) | -9.9 | (-32.3,12.6) |
| p-trend | 0.80 | | 0.52 | | 0.61 | | 0.49 | |

Note: All β estimates and 95% confidence intervals are obtained from linear regression models. Confidence intervals that do not include 0 are in **bold**. Model 1 was adjusted for BMI Z-score, age at Tanner stage 4, and daily energy level (total kCal/day). Model 2 was adjusted for model 1 plus maternal education level, hours of daily television watching after school, dairy intake (g/day), meat intake (g/day) and waist circumference. βs represent absolute differences in outcomes in the second, third, or fourth quartile of SSB intake compared to the first (the reference category). Significant trends were evaluated by modeling the median SSB intake within quartiles as a continuous variable and presented as p-values.
